# Human landscape disturbance and wildlife gut microbiota: global knowledge gaps

**DOI:** 10.7717/peerj.20545

**Published:** 2026-01-12

**Authors:** Rocío Paleo-López, Carolina S. Ugarte, Camila J. Stuardo, Andrea X. Silva, Constanza Napolitano

**Affiliations:** 1 Laboratorio de Genética de la Conservación, Departamento de Ciencias Biológicas y Biodiversidad, Universidad de Los Lagos, Osorno, Región de Los Lagos, Chile; 2 Programa de Doctorado en Ciencias, mención Conservación y Manejo de Recursos Naturales, Universidad de Los Lagos, Puerto Montt, Región de Los Lagos, Chile; 3 AUSTRAL-omics, Vicerrectoría de Investigación, Desarrollo y Creación Artística, Universidad Austral de Chile, Valdivia, Región de Los Ríos, Chile; 4 Institute of Ecology and Biodiversity, Concepción, Región del Biobío, Chile; 5 Cape Horn International Center, Puerto Williams, Región de Magallanes y la Antártica Chilena, Chile

**Keywords:** Gut microbiota, Wildlife, Human landscape disturbance, Bacterial community, Conservation

## Abstract

Wildlife gut microbiota (GM) comprises a dynamic microbial community that plays a key role in host adaptation, ecological interactions and health. Human landscape disturbances (*e.g*., habitat loss and fragmentation) may alter the diversity and composition of wildlife GM. Therefore, it is important to understand whether these changes are driven by habitat loss, fragmentation per se, or a combination of fragmentation and additional disturbances (*e.g*., human activities, interaction with domestic animals). We reviewed recent literature (2013–2023) concerning the effects of human landscape disturbance on wildlife GM alpha diversity, focusing on studies employing quantitative or qualitative landscape metrics. Of 119 reviewed studies, 62.2% (*n* = 74) used some type of landscape metrics, 58% (*n* = 69) incorporated landscape disturbance as a variable in their analyses, and 49.5% (*n* = 59) reported significant differences in at least one alpha diversity index. Among studies on free-ranging wildlife that found significant differences in any alpha diversity index (*n* = 52), 69.2% (*n* = 36) employed landscape metrics, and 55.8% (*n* = 29) explicitly described the type of disturbance associated with changes in GM alpha diversity index, with higher values in less disturbed landscapes compared to more disturbed landscapes (binomial sign test; *p* = 0.04). With respect to host species exhibiting significant variations in their GM alpha diversity index due to human landscape disturbance, there is an overrepresentation of species classified as “Least Concern” and an underrepresentation of species from certain regions, particularly South America. Despite growing research interest in this field, the available studies remain insufficiently extensive to establish clear overall patterns and trends, both globally and across different taxonomic groups. This review identifies methodological and geographical biases and emphasizes the need for more comprehensive studies in this field, considering host species ecology and quantitative landscape metrics as a substantial contribution for predicting ecosystem-level responses and informing effective conservation efforts.

## Introduction

The gut microbiota (GM) represents a dynamic microbial community with fundamental implications for host adaptation, ecological interactions and health (McKenney et al., 2018; Henry et al., 2021). Comprising bacteria, archaea, fungi, viruses and protozoa, the GM plays a central role in multiple physiological functions, including digestion, nutrient absorption, detoxification, immune system regulation, and pathogen resistance. Consequently, the GM contributes to host life history and fitness (Macke et al., 2017; Wang et al., 2017; Barko et al., 2018; Moeller & Sanders, 2020). These microbial communities engage in intricate interactions with their hosts, influencing key aspects of adaptive responses to environmental changes. Therefore, the study of wildlife GM has recently obtained considerable attention, as host-microbiota interactions provide essential insight into biological and ecological processes (Macke et al., 2017).

The diversity and composition of the GM are shaped by a complex interaction of intrinsic and extrinsic factors. Intrinsic factors encompass host-specific characteristics, including host genetics background, age, sex, and life history traits (Grieneisen et al., 2021; Sadoughi et al., 2022; Yan et al., 2022; Park & Do, 2024). In contrast, extrinsic factors are related to the surrounding ecological context, including environmental conditions, dietary intake and anthropogenic disturbances (Baniel et al., 2021; San Juan et al., 2020; Trujillo et al., 2022). Among these extrinsic factors, human landscape disturbance, such as habitat loss, fragmentation and urbanization, have emerged as one of the major threats to wildlife populations (Hunter, 2007). Within this framework, the adaptive capacity of wildlife populations inhabiting disturbed landscapes is critical for their long-term persistence (Henry et al., 2021).

On a global scale, changes in land cover due to anthropogenic activities have been increasingly driven by forestry, agricultural expansion, livestock grazing, and the growth of rural and urban areas (Ma et al., 2024). These human landscape disturbances increase ecosystem vulnerability by altering biogeochemical cycles, reducing ecosystem services, limiting habitat availability for refuge, and reducing dietary resources, ultimately resulting in biodiversity loss (Metzger et al., 2006). Furthermore, the expansion of rural and urban areas increases spatial overlap between humans and wildlife, thereby exposing wild animals to domestic pathogens, environmental contaminants, and the consumption of processed foods, garbage, and agricultural crops (Ma et al., 2024).

Changes in dietary resources and human-provisioned food can reduce GM diversity (Gillman, McKenney & Lafferty, 2022), affecting the host’s ability to extract nutrients, synthesize essential metabolites and maintain immune homeostasis (David et al., 2014). Although low-quality food may serve as an alternative energy source, it often lacks the nutritional complexity required to sustain a balanced microbial community, leading to shifts in GM structure. While diet is a key mediator between habitat disturbance and changes in GM, it is not the only contributing factor (Trevelline et al., 2019). Other plausible mechanisms include chronic physiological stress from increased noise or predator presence, competition with domestic animals, exposure to anthropogenic pollutants such as antibiotics and heavy metals, and altered social or territorial dynamics affecting microbial transmission (Handy et al., 2023; Zhu, 2023). These factors may interact in complex ways, making the relationship between landscape disturbance and gut microbiota structure inherently multifactorial (Bennett et al., 2016; McManus et al., 2021).

Empirical studies across different taxa and regions have begun to elucidate these multifactorial interactions. For instance, habitat degradation has been linked to alterations in microbial composition and functional diversity in primates from the Global South. Black howler monkeys from Mexico and red colobus monkeys from Tanzania suffered alterations in GM composition, likely driven by dietary changes due to human landscape disturbance and habitat degradation (Amato et al., 2013, Barelli et al., 2015). In Panama, Heni et al. (2023) reported species-specific GM responses to similar types of landscape disturbance across three generalist rodent and marsupial species. Conversely, Fackelmann et al. (2021) showed that habitat fragmentation per se did not affect the GM of a generalist rodent species, suggesting that the effects of landscape disturbance may vary depending on species traits or ecological context. To date, only one systematic review has addressed this topic (Nguyen et al., 2024), synthesizing the effects of urbanization on the wildlife GM by analyzing a database of 13 studies, yet no clear directional pattern in microbiota responses were identified. Therefore, the effects and directionality of habitat loss and fragmentation on wildlife GM remains understudied, and it is still unclear whether observed effects reflect species-specific responses or broader patterns across taxonomic groups (Alberdi, Martin Bideguren & Aizpurua, 2021). Additionally, few studies incorporate fine-scale, detailed landscape metrics, defined as quantitative landscape variables measured around sampling sites (areas biologically relevant for host ecology and disturbance context), such as habitat connectivity, patch number or size, or disturbance gradients. These metrics may capture local habitat heterogeneity that directly influences individual-level processes, allowing for more precise analyses when assessing relationships between GM and landscape variables.

Integrating fine-scale landscape metrics into microbiota analyses could substantially improve our understanding of how landscape configuration shapes gut microbial communities, with downstream effects on host health and adaptive potential. Beyond individual-level effects, changes in GM may have broader ecological consequences. Altered microbial communities may affect host adaptive potential and fitness traits, such as reproduction, dispersal ability, or disease resistance, ultimately influencing population dynamics and species interactions. Therefore, understanding how environmental disturbance alters gut microbiota composition is crucial for predicting ecosystem-level responses and informing conservation strategies (Hauffe & Barelli, 2019; Decaestecker et al., 2024).

Research on the effects of human landscape disturbance on GM is essential to improve species conservation strategies in environments under constant selective pressures, providing insights into the factors that contribute to species long-term persistence (Trevelline et al., 2019). This relevance is especially pronounced in biodiversity hotspots (*e.g*., Earth’s most biologically rich yet threatened terrestrial regions), where conservation efforts are urgently needed (Myers et al., 2000, Weinzettel, Vačkář & Medková, 2018). Most of these biodiversity hotspots are located in the Global South, including Central and South America, Africa, and Southeast Asia, with notable areas such as the Tropical Andes, Atlantic Forest, Mesoamerica, the Caribbean Islands, and Sundaland, among others (Hrdina & Romportl, 2017). These areas play a critical role in maintaining ecological resilience and providing essential ecosystem services due to their rich and diverse habitats. However, the Global South faces significant threats from habitat loss and fragmentation driven by urban expansion, agricultural practices, and natural resource extraction (Brooks et al., 2002). Despite harboring the majority of the planet’s terrestrial biodiversity, countries in the Global South remain significantly underrepresented in ecological and microbiome research (Skaldina & Blande, 2025). This geographic and taxonomic bias limits our ability to detect global patterns and to understand how microbiota-mediated processes respond to anthropogenic pressures in the most ecologically sensitive regions. Addressing this imbalance is crucial to inform context-specific conservation strategies and to ensure that global microbiome science is inclusive and representative.

To address these knowledge gaps, we conducted a systematic review synthetizing the literature on the effects of human landscape disturbance on wildlife GM diversity, focusing on bacterial communities assessed through 16S rRNA sequencing. Our hypothesis predicts that species inhabiting less disturbed landscapes exhibit higher alpha diversity in their GM compared to those in more disturbed landscapes (*e.g*., habitat loss and fragmentation). We evaluated this hypothesis by synthesizing studies that reported GM alpha diversity across sampling sites with more or less human landscape disturbance. Additionally, we explored whether these effects are associated with the host’s International Union for Conservation of Nature (IUCN) conservation status, as species classified under higher threat categories may be more vulnerable to human landscape disturbance (Betts et al., 2017). To address this, we categorized the reviewed studies based on to the IUCN conservation status of their focal host species. Finally, this review aims to identify current knowledge gaps regarding wildlife GM bacterial component response to human landscape disturbance, with a special focus on conservation priorities in the Global South.

## Survey methodology

**Figure 1:**
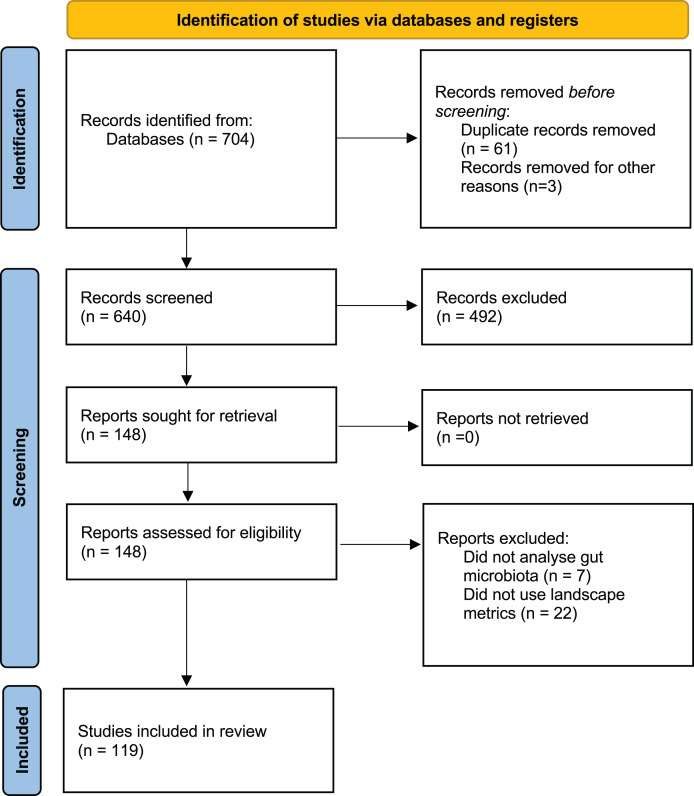
PRISMA flow diagram showing the selection of studies published between 2013 and 2023.

A comprehensive literature search was performed using Web of Science (WoS) and Google Scholar for peer-reviewed publications between 1 January 2013 and 10 April 2023 addressing the effects of human landscape disturbance on wildlife GM alpha diversity. The following search terms were used: (“Microbiota” OR “microbiome”) AND (“wild*”) AND (“habitat” OR “fragmentation” OR “landscape” OR “land-use” OR “anthropization” OR “perturbation” OR “habitat loss” OR “degradation” OR “deforestation” OR “land-cover” OR “land-use change”). The literature search and selection process (Fig. 1) was conducted following the PRISMA guidelines (Page et al., 2021). Two authors (RP-L and CSU) independently conducted the search strategy, and a third author (CJS) resolved any discrepancy.

The inclusion criteria for the selected published articles were as follows: (1) the studies must focus on free-ranging wildlife and/or captive wildlife species; (2) GM diversity must be assessed using molecular techniques; (3) human landscape disturbance (*e.g*., urbanization, fragmentation, habitat loss) must be evaluated using quantitative or qualitative landscape metrics; and (4) the study must explicitly indicate the relationship between landscape metrics and disturbance. Studies that did not directly assess disturbance but instead used keywords such as “disturbance”, “human disturbance,” “pollution”, or “human presence”, that allowed inference of landscape disturbance effects were also included. Additionally, studies incorporating landscape metrics in the statistical analysis were also considered eligible. Review articles and meta-analysis were excluded.

The preliminary search yielded a total of 704 articles, with 640 remaining after duplicates between databases were removed (*n* = 61). After screening titles and abstracts, 148 articles were retained. Of these, 29 articles were excluded because they did not analyze wildlife gut microbiota or lacked qualitative or quantitative landscape metrics. Finally, 119 articles were selected for data extraction process (Fig. 1; Table S1).

For each selected study, the following general data were recorded: (a) year of publication; (b) continent, region; (c) country; (d) host species; (e) IUCN conservation status of the host species (if not reported in the article, it was obtained from the IUCN red list web site (IUCN, 2024)); and (f) sample size (Table S1).

The GM data extraction included variables that could differ between studies and that were relevant for the comparison and replication of methods: (a) sample type (*e.g*., feces, swab); (b) sequencing platforms; (c) targeted amplicon region; (d) reported alpha diversity indices; (e) presence of significant differences in alpha diversity indices due to landscape/habitat metrics (Yes/No); and (f) direction of the effect of statistically significant differences in alpha diversity indices due to landscape/habitat metrics.

Landscape metrics data extracted included: (a) qualitative metrics (*e.g*., habitat types, urban/rural classification); (b) quantitative metrics (*e.g*., percent land cover); and (c) incorporation of landscape metrics in GM statistical analysis (Yes/No).

To explore tendencies in the effects of human landscape disturbance on alpha diversity indices, we focused exclusively on free-ranging wildlife because captive animals do not inhabit disturbed landscapes and are fed and maintained in human-controlled environments which have been described to alter their natural microbiota composition (Borbón-García et al., 2017; Eisenhofer, Helgen & Taggart, 2021). Among these studies, we classified those reporting significant differences in any alpha diversity indices separately from those reporting no significant differences. When studies included multiple species, each species was treated as a separate study case. To analyze whether a common pattern in the direction of change in the alpha diversity indices exists, we created a subset of free-ranging wildlife studies reporting categorical landscape metrics and significant differences in host GM. Then we classified the landscapes into two categories: (i) more disturbed landscapes (human-dominated) and (ii) less disturbed landscapes (natural habitats with minimal or no human disturbance). We performed a binomial sign test to assess the significance of these effects in the subset of free-ranging wildlife study cases. All analyses and graphs were conducted in R version 4.4.1 (R Core Team, 2024).

## Results

### General description of studies

**Figure 2:**
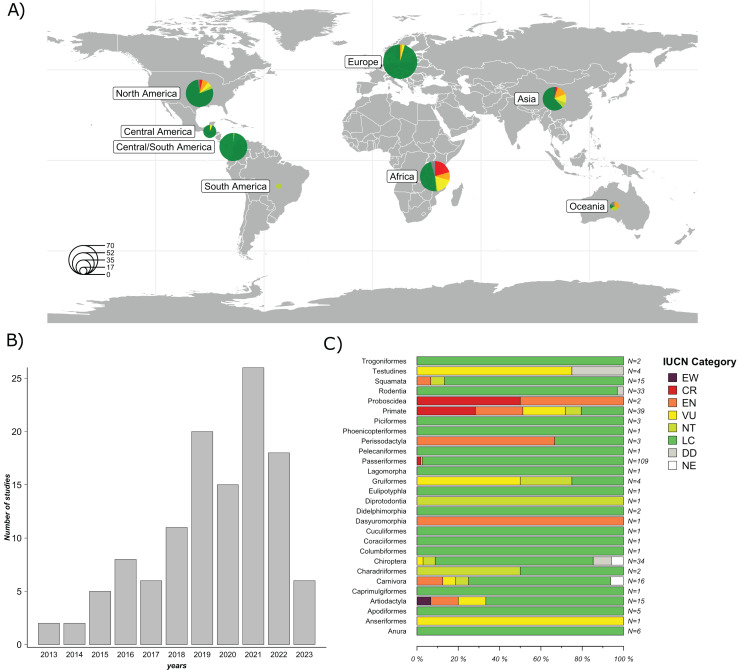
Overview of the studies evaluated: Geographic distribution of the species studied; Frequency of GM studies published per year; and Number of study cases per taxonomic order across all studies. (A) Geographical distribution of host species studied in the 119 articles evaluated. Circle size represents the number of host species studied per region. (B) Frequency of GM studies in wildlife published per year. The height of each bar represents the number of studies published annually. (C) Number of study cases per taxonomic order across all included studies. Each horizontal bar represents the proportion of species within an order classified under different IUCN conservation status categories: EW (Extinct in the Wild), CR (Critically Endangered), EN (Endangered), VU (Vulnerable), NT (Near Threatened), LC (Least Concern), DD (Data Deficient), and NE (Not Evaluated). N indicates the total number of species included in each order. Map was generated using the R package “rnaturalearthdata” (South, Michael & Massicotte, 2024).

A total of 119 studies published between January 1, 2013 and April 10, 2023, were included in the final evaluation (Fig. 2B). 58 studies (48.7%) were conducted in countries from the Global North, while 61 (51.3%) were conducted in countries from the Global South. The majority of the studies were conducted in North America (25.2%), followed by Asia (23.5%), Europe (21%), and Africa (17.7%). The least represented regions were Central America (5.9%), Oceania (5.0%), and South America (1.7%) (Fig. 2A). The complete list and metadata of the 119 studies included in this review are provided in Table S1.

A significant proportion of these studies (84%) focused on free-ranging (non-captive) wildlife, while a minor proportion focused on captive wild animals (16%). In terms of taxonomic representation, 21 orders were reported. The five most frequently reported orders were Primates (26%), Rodentia (14.8%), Carnivora (13.3%), Artiodactyla (12.3%) and Passeriformes (12.1%). The most commonly used method to characterize the GM was 16S rRNA gene sequencing by metabarcoding (99%). Of this gene, the most frequently targeted region was V4 (44.5%), followed by V3-V4 (29.4%); other regions accounted for 21.8%, and 3.3% of the studies did not specify the 16S rRNA region used. Regarding sample type, collected feces were the most frequently used (74.8%), followed by intestines or feces from carcasses (16%), rectal swabs (7.6%) and other types of samples (*e.g*., nest swab) (1.7%). Of the studies that used fecal samples, 95.5% reported using fresh samples (*e.g*., collected after defecation or in the field that had an evident mucous layer on the surface of the feces), while 4.5% did not mention sample freshness.

Regarding the species analyzed, 79.7% of the articles evaluated only one species, 15.2% evaluated between two and ten species, and 5.1% evaluated more than ten species, totaling 303 species studied. According to the IUCN conservation status (IUCN, 2024), species were categorized as follows: 77.9% Least Concern (LC); 5.9% Vulnerable (VU); 5.9% Endangered (EN); 4.6% Critically Endangered (CR); 3.6% Near threatened (NT); 1.7% Data Deficient (DD); and 0.3% Extinct in the Wild (EW). Among the species classified as EN (*n* = 18), nine belong to the order Primates, while the remaining orders include Artiodactyla (*n* = 2), Carnivora (*n* = 2), Dasyuromorphia (*n* = 1), Perissodactyla (*n* = 2), Proboscidea (*n* = 1), and Squamata (*n* = 1). Of the species classified as VU (*n* = 18), eight belong to the order Primates, while the remaining species belong to the orders Testudines (*n* = 3), Gruiformes (*n* = 2), Chiroptera (*n* = 1), Carnivora (*n* = 1), Artiodactyla (*n* = 2), and Anseriformes (*n* = 1). Among species classified as CR (*n* = 14), 11 belong to the order Primates, two to the order Passeriformes and one to the order Proboscidea. Among species categorized as EW, only one species from the order Artiodactyla (*n* = 1) was represented (Fig. 2C).

Regarding the use of landscape metrics in the studies, 62% (*n* = 74) used some type of landscape metrics. Most of them employed qualitative metrics (82.4%, *n* = 61), followed by quantitative metrics (9.4%, *n* = 7), and a combination of both (8.1%, *n* = 6). Of these studies, 85.1% (*n* = 63) explicitly linked the metrics to landscape disturbance, whereas ten studies did not establish a clear connection between the metrics and disturbance, and one did not relate the metrics to disturbance at all. Furthermore, 93.2% (*n* = 69) of the studies incorporated landscape disturbance as a variable in their analyses. Among studies using qualitative metrics, the majority (85.2%, *n* = 52) employed only two landscape categories, such as rural-urban or forest-farmland, while the remaining (14.8%, *n* = 9) used between three and nine categories. All studies analyzing quantitative metrics used some type of land cover data, such as tree cover percentage, human footprint score, or fragment size.

In relation to the GM alpha diversity index, 49.5% (59/119) of the total studies reported significant differences in at least one alpha diversity index across habitats, while 42.8% (*n* = 51) found no substantial changes in alpha diversity, and 7.7% (*n* = 8) reported changes in alpha diversity due to other factors. Regarding the use of different alpha diversity indices, among the studies employing landscape metrics (*n* = 74), 24.3% (*n* = 18) used a single index, 60.8% (*n* = 45) used two to three indices, 10.8% (*n* = 8) used more than three indices, and 4.1% (*n* = 3) did not estimate alpha diversity. The most frequently used index was Shannon (33.9%), followed by Phylogenetic Diversity (PD) (15.4%), Chao1 (13.1%), Observed Operational Taxonomic Units (OTUs) (12.5%), Observed Amplicon Sequence Variants (ASVs) (8.9%), Simpson (7.7%), and others (8.3%; *e.g*., Evenness, Abundance-based Coverage Estimator (ACE)). Of the studies on free-ranging wildlife that found significant differences in any alpha diversity index (*n* = 52), 69.2% (*n* = 36) used landscape metrics, while 55.8% (*n* = 29) explicitly described the categorical type of disturbance associated with changes in GM diversity indices.

**Figure 3:**
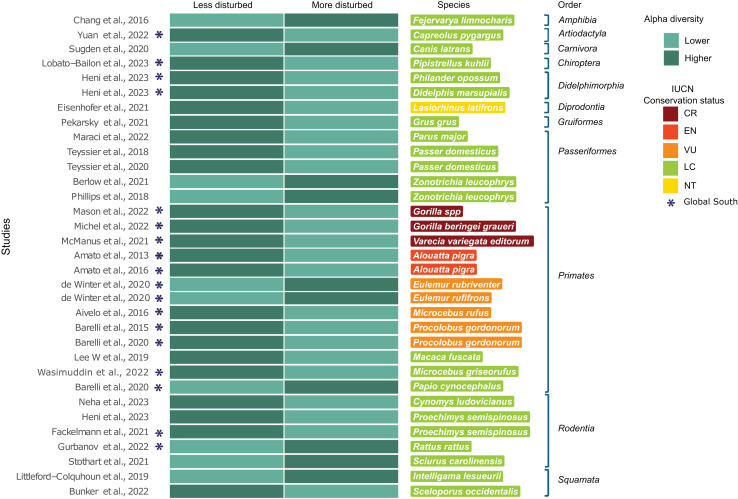
Subset of 29 from the 119 studies using landscape metrics that reported significant differences in any alpha diversity indices. Subset studies using landscape metrics that reported significant differences in any alpha diversity indices (*n* = 29), corresponding to 33 study cases, grouped by host taxonomic rank. (*) Represents studies conducted in Global South countries. The heatmap compares alpha diversity indices between less disturbed and more disturbed landscapes (see Methods), with darker colors representing higher alpha diversity indices and lighter colors representing lower alpha diversity indices. Host species are listed with colors indicating their corresponding IUCN conservation status category (Chang et al., 2016; Yuan et al., 2022; Sugden et al., 2020; Lobato-Bailón et al., 2023; Heni et al., 2023; Eisenhofer, Helgen & Taggart, 2021; Pekarsky et al., 2021; Maraci et al., 2022; Teyssier et al., 2018; Teyssier et al., 2020; Berlow, Phillips & Derryberry, 2021; Phillips, Berlow & Derryberry, 2018; Mason et al., 2022; Michel et al., 2022; McManus et al., 2021; Amato et al., 2013, 2016; Winter et al., 2020; Aivelo, Laakkonen & Jernvall, 2016; Barelli et al., 2015, 2020; Lee et al., 2019; Wasimuddin et al., 2022; Barelli et al., 2020; Neha & Salazar-Bravo, 2023; Fackelmann et al., 2021; Gurbanov, Kabaoğlu & Yağcı, 2022; Stothart & Newman, 2021; Littleford-Colquhoun et al., 2019; Bunker & Weiss, 2022).

A subset of 29 of the 119 studies (24.4%) included landscape metrics classified into categorizable landscape types (more/less disturbed) and reported significant differences in at least one alpha diversity index (Table 1). When studies included multiple species, each species was treated as a separate study case, encompassing a total of 33 study cases. Of the 29 studies, 48.3% (*n* = 14) were conducted in countries of the Global South. This subset (*n* = 29) included 28 host species, the majority of which (*n* = 19) are classified as LC. Eight of these species are from Global South countries, while the remaining 11 species are from countries in the Global North. Regarding threatened species (*n* = 8), all of them are from the Global South and belong exclusively to the order Primates, with three classified as CR, one as EN and four as VU. Of the subset, the only species classified as NT was a marsupial from the order Diprotodontia inhabiting in the Global North (Fig. 3). Sixty-six percent (*n* = 22) of the study cases reported higher alpha diversity values in less disturbed landscapes, while 33.3% (*n* = 11) reported higher alpha diversity values in more disturbed landscapes (exact binomial test; *p* = 0.04) (Fig. 3).

**Table 1:** Subset of studies (*n* = 29) that used landscape metrics and reported significant differences in any of the alpha diversity indices. (*) represents studies conducted in Global South countries. NM, not mentioned in the study. (+) indicates higher alpha diversity and (−) indicates lower alpha diversity.

Study	Order	Species	IUCN conservation status	Country	Taxonomic classification method	16S rRNA region	Less disturbed	More disturbed	Less disturbed results	More disturbed results	Alpha diversity Index
Amato et al. (2013)*	Primates	*Alouatta pigra*	EN	Mexico	OTU	V1–V3	Continuous forest	Fragmented forest	(+)	(−)	Chao1
Barelli et al. (2015)*	Primates	*Procolobus gordonorum*	VU	Tanzania	OTU	V1–V3	Continuous forest	Fragmented forest	(+)	(−)	Observed OTUs, Shannon, Chao1
Phillips, Berlow & Derryberry (2018)	Passeriformes	*Zonotrichia leucophrys*	LC	USA	OTU	V4	Rural	Urban	(-)	(+)	Shannon
Amato et al. (2016)*	Primates	*Alouatta pigra*	EN	Mexico−Costa Rica	OTU	V4	Continuous forest	Fragmented forest	(+)	(−)	Chao1
Winter et al. (2020)	Primates	*Eulemur rufifrons*	VU	Madagascar	OTU	V1–V2	National Park (less intensely logged site)	National Park (more intensely logged site)	(−)	(+)	Phylogenetic diversity
	Primates	*Eulemur rubriventer*	VU								Phylogenetic diversity
Aivelo, Laakkonen & Jernvall (2016)*	Primates	*Microcebus rufus*	VU	Madagascar	OTU	V1–V2	National Park	Degraded area	(+)	(−)	Richness, Inversed Simpson
Chang et al. (2016)	Amphibia	*Fejervarya limnocharis*	LC	Taiwan	OTU	V4	Natural habitat	Farmlands	(−)	(+)	Shannon, Chao1,Simpson, ACE
Teyssier et al. (2018)	Passeriformes	*Passer domesticus*	LC	Belgium	OTU	V5–V6	Rural	Urban	(+)	(−)	Observed OTUs
Littleford-Colquhoun et al. (2019)	Squamata	*Intelligama lesueurii*	LC	Australia	OTU	V3–V4	Isolated native habitat	City	(−)	(+)	Observed OTUs, Shannon
Lee et al. (2019)	Primates	*Macaca fuscata*	LC	Japan	OTU	V3–V4	Wild highland and lowland	Less provisioned; Crop-raiding; Intensively provisioned sites	(+)	(−)	Shannon
Barelli et al. (2020)*	Primates	*Papio cynocephalus*	LC	Tanzania	ASV	V1–V2	Continuous forest	Fragmented forest	(−)	(+)	Shannon, SV Number
	Primates	*Procolobus gordonorum*	VU				Continuous forest	Fragmented forest	(+)	(−)	Shannon
Teyssier et al. (2020)	Passeriformes	*Passer domesticus*	LC	France	OTU	V5–V6	Rural	Urban	(+)	(−)	Observed OTUs, Chao1
Sugden et al. (2020)	Carnivora	*Canis latrans*	LC	Canada	ASV	V4	Rural	Urban	(−)	(+)	Shannon, Phylogenetic diversity
Berlow, Phillips & Derryberry (2021)	Passeriformes	*Zonotrichia leucophrys*	LC	USA	OTU	V4	Rural	Urban	(−)	(+)	Bacterial richness
Stothart & Newman (2021)	Rodentia	*Sciurus carolinensis*	LC	Canada	OTU	V4	Rural	Urban	(−)	(+)	Observed OTUs
Pekarsky et al. (2021)*	Gruiformes	*Grus grus*	LC	Russia-Israel	ASV	V4	Areas with low human activities and higher proportion of non-cultivated land	Areas with higher proportion of cultivated and intensive agriculture	(+)	(−)	Phylogenetic diversity
McManus et al. (2021)*	Primates	*Varecia variegata editorum*	CR	Madagascar	ASV	V4–V5	Primary forest	Disturbed forest	(+)	(−)	Shannon, Phylogenetic diversity
Fackelmann et al. (2021)*	Rodentia	*Proechimys semispinosus*	LC	Panama	ASV	V4	Continuous forests	Fragmented forest	(+)	(−)	Observed ASVs, Shannon, Phylogenetic diversity
Eisenhofer, Helgen & Taggart (2021)	Diprotodontia	*Lasiorhinus latifrons*	NT	Australia	OTU	V4	Native grassland habitat	Remnant eucalypt woodland	(+)	(−)	Phylogenetic diversity
Michel et al. (2022)*	Primates	*Gorilla beringei graueri*	CR	Congo	ASV	V4	National Park (no human presence)	National Park (with human presence)	(+)	(−)	Observed ASV, Evenness
Bunker & Weiss (2022)	Squamata	*Sceloporus occidentalis*	LC	USA	ASV	V4	Forest	Beach	(+)	(−)	Shannon, Richness, Phylogenetic diversity
Yuan et al. (2022)*	Artiodactyla	*Capreolus pygargus*	LC	China	OTU	V3–V4	Continuous Forest	Plantation Forest	(+)	(−)	Observed OTUs, Shannon, Simpson
Mason et al. (2022)*	Primates	*Gorilla spp*	CR	Congo	ASV	V3–V4	National Park	Periphery of National Park	(+)	(-)	Observed ASV
Maraci et al. (2022)	Passeriformes	*Parus major*	LC	Poland	OTU	V3–V4	Rural	Urban	(+)	(−)	Phylogenetic diversity
Gurbanov, Kabaoğlu & Yağcı (2022)*	Rodentia	*Rattus rattus*	LC	Turkey	OTU	V3–V4	Rural	Urban	(−)	(+)	Shannon, Simpson
Wasimuddin et al. (2022)*	Primates	*Microcebus griseorufus*	LC	Madagascar	ASV	V4	Continuous dry spiny forest	Forest remnants and agricultural fields	(+)	(−)	Shannon, Phylogenetic diversity, Observed species
Neha & Salazar-Bravo (2023)	Rodentia	*Cynomys ludovicianus*	LC	USA	OTU	V1–V3	Rural	Urban	(+)	(−)	Observed OTUs, Shannon, Phylogenetic diversity
Lobato-Bailón et al. (2023)*	Chiroptera	*Pipistrellus kuhlii*	LC	Spain	NM	16S gene	Mature and immature forest	Farming and Urban	(+)	(−)	Shannon
Heni et al. (2023)*	Rodentia	*Proechimys semispinosus*	LC	Panama	ASV	V4	Continuous forest	Fragmented forest	(+)	(−)	Shannon, Phylogenetic diversity
	Didelphimorphia	*Didelphis marsupialis*	LC								
	Didelphimorphia	*Philander opossum*	LC								

## Discussion

This review provides a novel and comprehensive synthesis of the effects of human landscape disturbance on the diversity of wildlife gut microbiota (GM) at a global scale, with a focus on bacterial communities. Its scope encompasses the use of both qualitative and quantitative landscape metrics, and the conservation status of the host species studied. The main sample type and gene sequenced for studying wildlife GM were fresh feces and the V4 region (16S rRNA). Representation from both the Global South and the Global North was balanced, with species classified as Least Concern being most commonly studied, and Primates being the predominant order among threatened species examined. Half of the studies reviewed reported significant differences in GM alpha diversity across different levels of human landscape disturbance, and 62% of the studies used some kind of landscape metrics. Among study cases in which GM alpha diversity and landscape disturbance were correlated, GM diversity tended to be negatively associated with human landscape disturbance. We suggest that employing standardized landscape metrics will facilitate GM comparisons across studies, enabling robust general conclusions.

Studies utilizing fecal samples confer a clear methodological advantage, as feces represent an environmental sample that can be collected through a non-invasive method. This approach allows for the inclusion of a wide range of species, including elusive and threatened animals such as carnivores, which exhibit low density populations and play pivotal roles in ecological balance (Roemer, Gompper & Valkenburgh, 2009). Fecal sampling eliminates the need for animal capture, thereby minimizing animal disturbance and stress, while also reducing logistical complexity and expenses (De Flamingh et al., 2023). Most reviewed studies used fresh fecal samples for GM analysis. The freshness of collected feces is a crucial consideration when evaluating GM, as its quality is susceptible to environmental factors, especially temperature and humidity. These factors influence sample preservation and the accuracy of GM diversity assessments (Combrink et al., 2023). Additionally, the challenge of determining the precise time of fecal deposition adds complexity, as prolonged environmental exposure can modify microbial communities (Menke, Meier & Sommer, 2015). Therefore, maintaining optimal sample preservation conditions during collection and transport (*e.g*., >95% ethanol, buffer solution, freeze sample) is essential for ensuring robust and reliable GM analyses (Combrink et al., 2023). Sampling fresh feces can foster the expansion of GM research including underrepresented taxonomic groups, thereby providing a more comprehensive picture of how human landscape disturbance influences host GM. This knowledge offers valuable insights into species’ digestive health and their adaptive potential to disturbed environments (Henry et al., 2021), which are important considerations for biodiversity conservation.

Regarding the methodologies used to evaluate wildlife GM diversity, the reviewed studies employed different taxonomic classification methods (*e.g*., OTUs or ASVs) and targeted different regions of the 16S rRNA gene. The selection of taxonomic classification methods has a significant impact on alpha diversity estimates (Chiarello et al., 2022). Similarly, the choice of 16S rRNA gene regions targeted for high-throughput sequencing can influence the portion of microbial diversity captured, as different regions vary in their taxonomic level resolution capabilities (Sikolenko & Valentovich, 2022). While the V3–V4 region is commonly used due to its balance between taxonomic breadth and resolution, other regions such as V5–V6 may offer more limited coverage across bacterial phyla (Sikolenko & Valentovich, 2022; Weinroth et al., 2022). Consequently, methodological variations and differences in targeted 16S rRNA gene regions may lead to discrepancies in alpha diversity estimates, hindering comparisons between studies. Additionally, evaluating rarefaction curves is crucial to ensure sufficient sequencing depth to accurately reflect the GM diversity within samples, facilitating standardization of sequencing effort—a key consideration in ecological studies where sequencing depth often varies (Weiss et al., 2017). Without this step, some of the differences might be due to technical biases and not to actual biological variation (Cameron et al., 2021). The employment of consistent methodologies and the judicious consideration of 16S rRNA gene regions selection are imperative for reliable interpretations and robust comparisons of the impacts of human landscape disturbance on wildlife GM diversity.

The choice of alpha diversity index (*e.g*., Shannon, Simpson, Chao1, Evenness) may influence the interpretation of results and might introduce discrepancies in comparisons between studies (Kers & Saccenti, 2022). Therefore, when evaluating the effects of human landscape disturbance on wildlife GM diversity, it is crucial to consider how these methodological decisions influence the interpretation of reported alpha diversity values. Despite Shannon index was the most frequently used, it was reported in only one third of the studies. We recommend that future studies systematically report all indices used, including non-significant outcomes, along with their associated statistical values (*e.g*., mean, standard deviation), including effect size and sample sizes. This practice would improve results transparency and completeness, facilitate retrospective analysis of GM studies, support the development of meta-analyses, and enable reassessments of statistical findings (Kambach et al., 2020; Kers & Saccenti, 2022).

In relation to landscape metrics, we identified a gap in the standardization of how “disturbed landscapes” are defined and measured. Many studies define “disturbance” based on qualitative descriptions or broad classifications (*e.g*., natural forest/plantation forest or rural/urban), often without clear criteria, limiting cross-study comparability and hindering the understanding of how human disturbance affects wildlife GM along a landscape gradient. Moreover, only a minority of studies employed quantitative metrics, which offer a more objective assessment of human landscape disturbance. However, the limited number of studies precluded a comprehensive quantitative analysis of GM diversity. To illustrate the use of quantitative metrics use in such analyses, Phillips, Berlow & Derryberry (2018) evaluated the effect of habitat urbanization on the GM of *Zonotrichia leucophrys* by analyzing the percentage of several land cover types (impervious, tree, shrub, grass). This approach aims to identify which landscape disturbance factor drives the diversity of GM. Different disturbance types, such as habitat fragmentation, habitat loss, or contamination, can exert distinct effects on GM (Fackelmann et al., 2021), highlighting the importance of adopting rigorous, standardized methods for characterizing human landscape disturbance, diminishing challenges in cross-study comparisons. Therefore, establishing clear and consistent metrics is crucial to accurately determine whether changes in GM diversity are attributable to human landscape disturbance (habitat loss, fragmentation), contamination, additional anthropogenic disturbances (*e.g*. contact with humans, domestic animals, exposure to pathogens) or the combination of these.

Human landscape disturbance and GM diversity might be related and could impact wildlife health (Barelli et al., 2015). Our analysis of the direction of change in the GM alpha diversity indices relative to human landscape disturbance aligned with our hypothesis. This trend supports the statement that human landscape disturbance (*e.g*., habitat loss and fragmentation) can affect wildlife hosts and alter their gut microbial community structures. It is important to consider that these effects may be interacting with host-specific ecological traits, which can be addressed by a multifactorial approach. Including key ecological traits, such as whether species are habitat or dietary generalists or specialists, may provide a more comprehensive understanding of human landscape disturbance effects. This distinction is important because a species’ sensitivity to landscape disturbance directly influences its foraging behavior, habitat use, and exposure to novel pathogens (Brearley et al., 2013; Gillman, McKenney & Lafferty, 2022; Burton et al., 2024). Knowledge on host species ecology may contribute with critical context for interpreting the effect of human landscape disturbance on different hosts’ GM and should be considered as relevant information when interpreting results.

Most species assessed in the reviewed studies were classified as Least Concern (LC) according to the IUCN Red List. In contrast, species categorized as Vulnerable, Endangered or Critically Endangered (VU, EN or CR) were markedly underrepresented. This pattern likely reflects the higher population abundances of the LC species, which facilitates sample collection and enhances the project feasibility. However, this sampling bias in wildlife GM research contributes to significant knowledge gaps, as species vary in their ecological traits, dietary flexibility, and sensitivity to habitat alteration (Barelli et al., 2020; Burton et al., 2024). Threatened species, in particular, stand to benefit most from microbiome studies, which can inform conservation efforts, yet remain understudied. Additionally, a clear taxonomic bias toward primates was evident. Expanding future research to encompass a broader range of taxonomic groups and increasing attention to underrepresented regions–such as South America, which harbors high biodiversity but has limited microbiome studies incorporating landscape disturbance variables–would provide valuable contributions to the field. Broadening both the taxonomic and regional scope in future research could reveal more generalizable patterns and enhance our overall understanding of how landscape disturbance impacts wildlife GM across species.

Addressing the key factors and methodological gaps identified in this review, such as: reporting all alpha diversity indices; selecting suitable 16S rRNA gene targeted regions; evaluating and applying appropriate rarefaction thresholds; clearly defining landscape metrics and properly associating them with GM diversity; and reporting comprehensive summary statistics to facilitate meta-analysis, will contribute significantly to advancing our understanding of wildlife GM. The implementation of these improvements will also guide future research efforts and enable more robust, comparable, and ecologically relevant findings.

## Conclusions

This review provides an overview of the effects of human landscape disturbance on the alpha diversity of wildlife bacterial GM. The identified methodological and geographical biases emphasize the need for more comprehensive studies in this subject. The GM plays a crucial role in host health and well-being by contributing to key processes like digestion, immune system modulation, and pathogen protection. Alterations in these microbial communities can negatively impact the health of their hosts, particularly those already threatened by habitat loss and fragmentation.

Promoting research in biodiversity-rich regions like South America is crucial, with a particular focus on threatened species inhabiting these vulnerable ecosystems. Furthermore, given the current taxonomic focus on specific groups, it is essential to diversify into less studied groups, such as carnivores. In this context, non-invasive fresh fecal sampling offers a great opportunity to expand the range of species studied by providing access to information on elusive species microbiota. This non-invasive approach not only facilitates the study of rare or protected species but also minimizes the impact of sampling on individuals of already vulnerable populations.

Establishing standardized methodologies, rigorously characterizing disturbed habitats, and considering ecological aspects of the host species are critical for enhancing cross-study comparisons and advancing our understanding of how human landscape disturbance impacts wildlife GM. Such improvements will enable the identification of broader patterns across taxonomic groups, ultimately informing the development of more effective conservation strategies that address the intricate relationship between GM and ecological health in the current global scenario of biodiversity loss.

## Supplemental Information

10.7717/peerj.20545/supp-1 Supplemental Information 1 Studies selected for data extraction (*n* = 119). Data from columns 6, 7, and 8 correspond to attributes of the host species evaluated in the studies

10.7717/peerj.20545/supp-2 Supplemental Information 2 PRISMA checklist.

## Additional Information and Declarations

### Data Availability

The following information was supplied regarding data availability:

The raw data is available in the Supplemental File.
